# Supplementary material for: Patterns of Intron Gain and Loss in Fungi
Source: PLoS Biol. 2004 Nov 30;2(12):e422. doi: 10.1371/journal.pbio.0020422 (PMC532390; doi:10.1371/journal.pbio.0020422)
Supplement: Table S1 — Also available at http://genes.mit.edu/NielsenEtAl/. (4.3 MB ZIP). [file pbio.0020422.st001.zip › NielsenEtAl/html/65.html]

AN0431.1.NCU03127.1.MG01324.1.FG00740.1


```
 CLUSTAL W (1.82) Multiple Sequence Alignments - Introns Inserted


Sequence 1: NCU03127.1	842 aa
Sequence 2: MG01324.1	835 aa
Sequence 3: FG00740.1	836 aa
Sequence 4: AN0431.1	1159 aa
Alignment Length: 1165 aa
Number Identitical Residues: 525 aa
Alignment Score (without introns) 24602


MG01324.1 	----------------------~-------------------------------------
NCU03127.1	----------------------~-------------------------------------
FG00740.1 	----------------------~-------------------------------------
AN0431.1  	MPKNDSKTVTIDVEEFTKTRDS0FLAKLAQLQSLTFELSRAYINHTSAVLGQDNANVDIS
          	 ....:.: : . .. :.: .:   :. :. .: : . : :  . :::  ....:. . :

MG01324.1 	------------------------------------------------------------
NCU03127.1	------------------------------------------------------------
FG00740.1 	------------------------------------------------------------
AN0431.1  	AITNTLAASLRDTGVLAAAGTGSGAESGEKKKRKRRADPNAPKRTLTPFFLYMHHNRARI
          	: :.: :::  .:.  :::.:.:.:.:..... .  :...:.. : :.       . :  

MG01324.1 	------------------------------~-----------------------------
NCU03127.1	------------------------------~-----------------------------
FG00740.1 	------------------------------~-----------------------------
AN0431.1  	AEELGPDAKPKDVSNEGTRRWAEMPDSQKE0VWKKLYADNLAAYREKVAAYKAGLPFDRD
          	:.. ...:.... :...:   :. ..:...   ..  :.. ::  .. :: .:. . . .

MG01324.1 	------------------------------------------------------------
NCU03127.1	------------------------------------------------------------
FG00740.1 	------------------------------------------------------------
AN0431.1  	DNDKAADQLHLDVAAAEASDEEEEEEEDEHGEEEEEEEEESPEPAKEPTPPPPKRRRSEG
          	....::..   . :::.::.......... ..........:...:...:.....   :..

MG01324.1 	---------------------------------------------------MH~LVPRE0
NCU03127.1	---------------------------------------------------MH~LIPKE0
FG00740.1 	---------------------------------------------------MH~LVPKE0
AN0431.1  	KPSKDVSSPVVEKKGRNESPEKKRRGAAKKDKEEPRKSLGGESKRSKKKRKSD0YIPIR~
          	..:.. ::.  .... ..:....  .::....... .: ...:. :... . .  :* . 

MG01324.1 	LDKLVITQL-----------------~-----------------G~HLAQRRLARGVKLN
NCU03127.1	LDKLAISQL-----------------~-----------------G~FLAQRRLARGIKLN
FG00740.1 	IDKLVISQL-----------------~-----------------G~ALAQRRLARGVKLN
AN0431.1  	EENWFASQLTILARRPFYFKYIPDII2KGRIHRQAWQYAPYSEGS1FLAQRRLARGVRLN
          	 ::   :**:  :  .   .  ..   ..    .: . :. :...  *********::**

MG01324.1 	HSEAT~ALITCVLQE~LIRDGAHTVSDLMSLGATMLGRRHVMPSVCSTLHEIQVEGTFPS
NCU03127.1	HSEAT0ALIANNLQE~LIRDGNHTVADLMALGATMLGRRHVLPEVTSTLHEIQVEGTFPS
FG00740.1 	HSEAV0ALIANNLHE~LIRDGNHTVSDLMALGATMLGRRHVMPSVCATLHEIQVEGTFPS
AN0431.1  	HAEAA0ALISSNLHE0LIRDGQYSVADLMSIGKTMLGRRHVLPSVPSTLVELQVEGTFTT
          	*:**. ***:  *:* ***** ::*:***::* ********:*.* :** *:******.:

MG01324.1 	GTYLVTVHNPIATDDGDLMRALYGSFLPVP--DADLFSMPELEDYEPERQPGAVVAVKG-
NCU03127.1	GTYLVTVHNPISSDDGDLARALYGSFLPIPPNDDSVFPLPPAAAYEASKQPGAVVCVKEA
FG00740.1 	GTYLVTVHNPISSDDGDLRRALYGSFLPVP--DNSIFPMAASEEYQLGKQPGAVIPVKTK
AN0431.1  	GSYLVTVHHPISSDDGDLEKALYGSFLPIP--PADTFPDPDPEDYLPEKVPGAVIPVKNA
          	*:******:**::***** :********:*    . *. .    *   : ****: **  

MG01324.1 	VVELNKGRRRRRLRVVSKGDRPVQVGSHYHFVEANAQLEFDRGKAYGFRLDIPAGTSVRF
NCU03127.1	KVKLNEGRKRIRLRVTSKGDRPIQVGSHYHFIETNPQLEFDRARAYGYRLDIPAGTSVRF
FG00740.1 	KITLNEGRKRIRLQVTSMGDRPIQVGSHYHFIETNPQLEFDRIRSYGYRLDIPAGTSVRF
AN0431.1  	RITLSEGRKRIKLKVMSKGDRPIQVGSHYHFIEANPQLHFDRFRAYGYRLDIPAGTSVRF
          	 : *.:**:* :*:* * ****:********:*:*.**.*** ::**:************

MG01324.1 	EPGDAKTVTIVEIGGSKAIRGGNWLANGDVDYSRVEEFIQSCQSQGFAHVP-APVWDDED
NCU03127.1	EPGDTKTVTLVEIGGNKVIRGGNNLASGKLDLTRAEEIIAKLQKAGFAHAPEEPVGDAAA
FG00740.1 	EPGDTKTVTLVEIGGKRVIRGGNNLASGVVDLSRADEILARLQEAGYAHKS-NPAGDMAH
AN0431.1  	EPGDTKTVTLVEIGGHRIIKGGNSLASGPVDLRRADDIIQRLQTAGFAHVP-EPAADNAL
          	****:****:***** : *:*** **.* :*  *.::::   *  *:** .  *. *   

MG01324.1 	--VKGFVLERNAYVTMFGPTTGDRVRLGMTDLWIEVERDMTVYGDECKFGGGKTLREGMG
NCU03127.1	FISEPFTMDRRAYAVMFGPTVGDMVRLGATDLWIKVEKDYTVYGDECKFGGGKTLREGMG
FG00740.1 	--IDPFQMDHASYATMFGPTTGDLVRLGSTDLWIKVERDETIYGDECKFGGGKTLREGMG
AN0431.1  	--VAPFTIDREAYARLFGPTTGDLIRLGLTNLWVKIEKDYTHYGDECSFGGGKSIREGMG
          	     * ::: :*. :****.** :*** *:**:::*:* * *****.*****::*****

MG01324.1 	QASGRSDEETLDMVVTNALILDWSGIYKADIGIKNGMIVAIGKAGNPDVMDGITEGMVVG
NCU03127.1	QATGRSDEETLDMVVTNALVVDWTGIYKADIGVKAGVIVGIGKAGNPDVMDGVTPGMIVG
FG00740.1 	QASGRTDAETLDLVVSNALIVDWTGIYKADIGIKEGMIVAIGKAGNPDVMDGVTEGMVVG
AN0431.1  	QASGKSHKDCLDTVITNAVIIDWSGIYKADIGIKNGTIVGIGKSGNPDVMDGVHPDMIIG
          	**:*::. : ** *::**:::**:********:* * **.***:********:  .*::*

MG01324.1 	SCTDVVAGEGKIITAGGIDTHIHFICPQQADEALAAGVTTFLGGGTGPS2AGTNATTCTP
NCU03127.1	SCTDVVAGEGKIITAGGIDTHIHFICPQQAAEALAAGITTFLGGGTGPS2AGTNATTCTP
FG00740.1 	SCTDVVAGEGKIVTAGAIDSHIHFICPQQVPEALASGVTTMLGGGTGPS2AGTNATTCTP
AN0431.1  	SSTDVIAGENKIVTAGGFDTHIHFICPQQAQEALASGITTFLGGGTGPS~TGTNATTCTP
          	*.***:***.**:***.:*:*********. ****:*:**:******** :*********

MG01324.1 	GKHYTKQMLQAADTLPLNIGITGKGNDSGLPGLRDQVISGQCGLKIHEDWGSTPAAIDAC
NCU03127.1	GAHYMRQMLQACDSLPLNIGITGKGNDSSPSALREQILAGACGLKLHEDWGTTPAAIDSC
FG00740.1 	GVHYMRSMLQACDQLPINIGITSKGNDSSPEGLRDQVNAGACGLKLHEDWGSTPAAIDTC
AN0431.1  	GPTHMRQMIQACDQIPINVGITGKGNDSGGIGIEEQIIAGAAGLKLHEDWGSTPAAIDTC
          	*  : :.*:**.* :*:*:***.*****.  .:.:*: :* .***:*****:******:*

MG01324.1 	LSVCEEFDVQCMIHTDTLNESGFVEATVAAFKGRTIHTYHTEGAGGGHAPDIIRVVEHDN
NCU03127.1	LTVCDELDVQCLIHTDTLNESGFVESTIAAFAGRTIHTYHTEGAGGGHAPDIISVVEHPN
FG00740.1 	LSVCDEFDIQCLIHTDTLNEAGFVESTIAAMKGRTIHTYHTEGAGGGHAPDIISVVEHEN
AN0431.1  	LDICEKYDVQCMIHTDTLNESGFVEQTIQAFKNRTIHTYHTEGAGGGHAPDIISVVEHPN
          	* :*:: *:**:********:**** *: *: .******************** **** *

MG01324.1 	VLPSSTNPTRPFTRNTLDEHLDMLMVCHHLSRDIPEDVAFAESRIRAETIAAEDVLHDLG
NCU03127.1	VLPSSTNPTRPFTRNTLDEHLDMLMVCHHLSKNIPEDVAFAESRIRAETIAAEDVLHDLG
FG00740.1 	VLPSSTNPTRPFTLNTLDEHLDMLMVCHHLSKNIPEDVAFAESRIRAETIAAEDVLHDKG
AN0431.1  	VLPSSTNPTRPFTMNTLDEHLDMLMVCHHLSKNIPEDVAFAESRIRAETIAAEDVLHDLG
          	************* *****************::************************* *

MG01324.1 	AISMMSSDSQAMGRVGEVVLRTWNTAHKNKVQRGRLPEDEANG-NDNCRAKRYVSKYTIN
NCU03127.1	AISMMSSDSQAMGRCGEVILRTWNTAHKNKLQRGPLPEDQGTGGADNHRVKRYISKYTIN
FG00740.1 	AISMMSSDSQAMGRCGEVILRTWNTAHKNKVQRGWLPEDEGTG-ADNARVKRYVSKYTIN
AN0431.1  	AISMMSSDSQAMGRCGEVILRTWNTAHKNKEQRGQLPEDENTG-ADNFRVKRYISKYTIN
          	************** ***:*********** *** ****: .*  ** *.***:******

MG01324.1 	PALAQGMSHLIGSVEVGKLADLVVWDPAWFGTKPNLVIKSGLIACAMM~GDPNASIPTIQ
NCU03127.1	PALAQGMSHLIGSIEPGKLADLVVWDPAWFGTKPSLVIKSGLIASAMM~GDPNASIPTVQ
FG00740.1 	PAIAQGFGHVIGSIEVGKFADLVLWDPAWFGTKPSYVLKGGHIAYAQM0GDPNASIPTVQ
AN0431.1  	PAIAQGMSHLIGSVEVGKLADLVIWSPSYFGTKPSQVLKSGMIVASMM0GDPNGSIPTIQ
          	**:***:.*:***:* **:****:*.*::*****. *:*.* *. : * ****.****:*

MG01324.1 	PIVARPMFA~PRVPQTSVLFVSQASVDQGVVQTYGLRKRVEPVKGCRSVGKKDMKHNAVM
NCU03127.1	PVIARPMFA~PMVAKSKVLFVSQASLDSGAIEGYGLKSRVEAVKGCRTVGKKDMKFNERM
FG00740.1 	PIIARPMFS~PHCASTSILFVSSASIETGAIASYGLRSRVEAVRGCRSIGKSDMKHNDTK
AN0431.1  	PVIMRPQFG0AYLPSTSVMFVSQASLDTNTVQSYGLKKRVEAVKNCRNIGKADMKFNDTM
          	*:: ** *. .  ..:.::***.**:: ..:  ***:.***.*:.**.:** ***.*   

MG01324.1 	PRMRVDAETYTVEADGVVCTALPSDELPLTQQYFVY
NCU03127.1	PRMKVDPERYTVEADGEVCTAEPSSELPLTQSYFVY
FG00740.1 	PRMRVDPESYTVEADGEICEAAPADTLPLTQQFYVY
AN0431.1  	PKMKVDPESYRVEADGRLCDAQPAETLPLTQDYFVY
          	*:*:**.* * ***** :* * *:. *****.::**
```
